# A Case Report of Spinal Arteriovenous Fistula: Vague Presentation and Successful Outcome

**DOI:** 10.7759/cureus.57017

**Published:** 2024-03-27

**Authors:** Sherif Eltawansy, Ghasan Ahmad, Neil Majmundar

**Affiliations:** 1 Internal Medicine, Jersey Shore University Medical Center, Neptune, USA; 2 Vascular Surgery, Jersey Shore University Medical Center, Neptune, USA

**Keywords:** neurovascular, angiogram, therapeutic embolisation, arteriovenous fistula repair, spinal deformiites

## Abstract

Spinal arteriovenous fistula (spinal AVF) malformation is one of the rare spinal vascular diseases. Its presentation could be misleading as the patient presents with spinal cord dysfunction, including motor power loss.

Early detection is essential and requires a high suspicion by the providing physician so the patient can be rightfully directed to the proper team with vascular intervention resources. Efficient management leads to promising outcomes with patient recovery.

We are presenting a case with progressing motor and sensory neurological deficits that had a vague clinical course. After a prompt diagnosis of spinal AVF, the patient was referred to the neuro-vascular specialist, who performed an embolization of the spinal AVF. The patient had an excellent outcome and was discharged to a rehabilitation facility.

## Introduction

Spinal vascular malformations (SVMs) comprise a rare subgroup of all spinal intradural lesions. It includes complex spinal arteriovenous malformations (AVMs) and arteriovenous fistulas (AVFs). That group could present with acute, subacute, or chronic spinal cord dysfunction. Failure to early recognize these lesions ends up with a permanent neurological deficit. It is one of the preventable spinal vascular diseases [1]. A spinal dural AVF (SDAVF) is an abnormal connection between radicular arteries and veins in the dura of an adjacent nerve root sleeve. The direct valveless arterial inflow into the coronal venous plexus raises its pressure. It causes venous congestion and intramedullary edema, a condition called congestive or venous hypertensive myelopathy. It also could lead to the possibility of spinal cord ischemia or infarction. Manifestations include sensory and motor deficits or sphincter dysfunction. Management usually occurs either by microsurgical or endovascular intervention [2].

## Case presentation

This is a case of a 62-year-old white female who presents with dizziness and nausea. She has a past medical history of hypertension, hyperlipidemia, right temporal lobe meningioma, non-Hodgkin lymphoma complicated with chemotherapy-induced neuropathy, intestinal lymphoma complicated with a stricture requiring manual dilation, esophageal spasm, and stricture post-dilation, type 2 diabetes mellitus, and anxiety.

**Figure 1:**
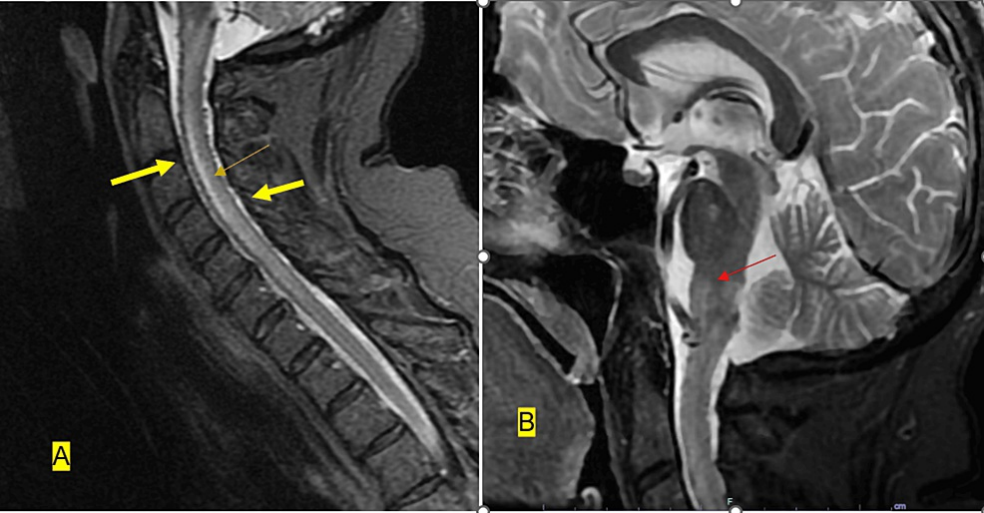
Cervical MRI with and without contrast Sagittal T2-weighted cervical MR demonstrating flow voids along (A) the ventral and dorsal surface of the medulla and cervical spinal cord (yellow arrows), linear cervical cord edema (orange arrow), and (B) edema within the pons and medulla (red arrow). As suspected on the prior MRI of the brain, there was a high T2 signal in the lower medulla and spinal cord, extending all the way down to the bottom of C6. It involved the posterior two-thirds of the cord most of the way, although, at the level of C2, it involved nearly the entire cross-section of the cord. On the sagittal T2 and STIR images, there was prominence of the posterior and anterior spinal arteries, which had a somewhat sawtooth appearance. Enhancement: There was a patchy enhancement in the cervical medullary junction and upper cord at the level of C1 seen on axial series 3 images 2 through 5 and post-contrast sagittal T1 image 7.

She had bilateral neuropathy at the feet from chemotherapy. However, she developed new numbness in her legs extending from her feet. It progressed to weakness in both lower extremities and left upper extremity. Vital signs on admission showed that the patient was afebrile, with a blood pressure of 127/74 mmHg and a pulse of 70 beats per minute. A neurological exam showed that she was awake and alert. Cranial nerves were intact, and facial sensations were intact. Motor power was 4/5 in the right upper extremity and 3/5 in the left upper extremity. Finger flexors on both sides were 4/5. Motor power in both lower extremities was 0/5 with diminished muscle tone. Sensation to light touch and coordination were intact in all extremities. No clear spinal cord sensory level is noted. Cerebellar testing showed intact finger-nose-finger testing. The patient denied back pain, urinary or bowel symptoms, or saddle anesthesia. Providers suspected acute stroke initially. Workup for stroke included CT head that came negative for acute findings. MRI of the brain without contrast showed signal abnormality at the medulla and upper cervical spinal cord, suggestive of an SDAVF. Cervical spinal MRI with and without contrast showed extensive high signals in the lower medulla and spinal cord down to the level of C6, suggestive of a spinal AVF (Figure 1).

**Figure 2:**
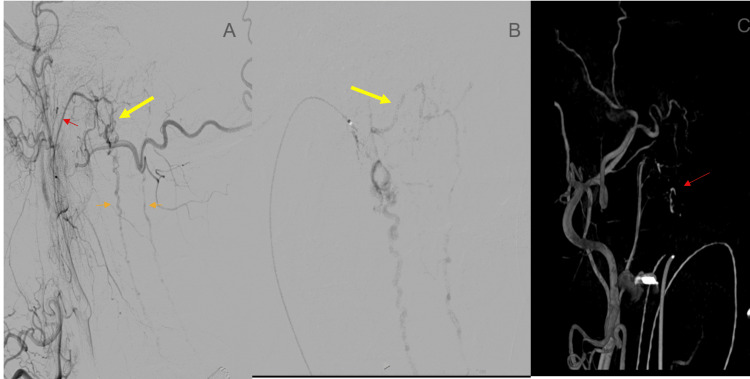
Cerebral and cervical spinal angiography (A) Right external carotid artery angiogram, lateral view demonstrating an arteriovenous shunting lesion centered at the premedullary cistern (yellow arrow) with drainage into the anterior and posterior spinal veins (orange arrows). Arterial supply is from the neuromeningeal trunk of the right ascending pharyngeal artery (red arrow). (B) Microcatheter angiogram of the right ascending pharyngeal artery, which confirms the site of the fistula. Of note is venous drainage into the transverse brainstem venous plexus (yellow arrow). (C) 3D rotational angiography in anteroposterior projection demonstrating the site of the fistula (red arrow).

**Figure 3:**
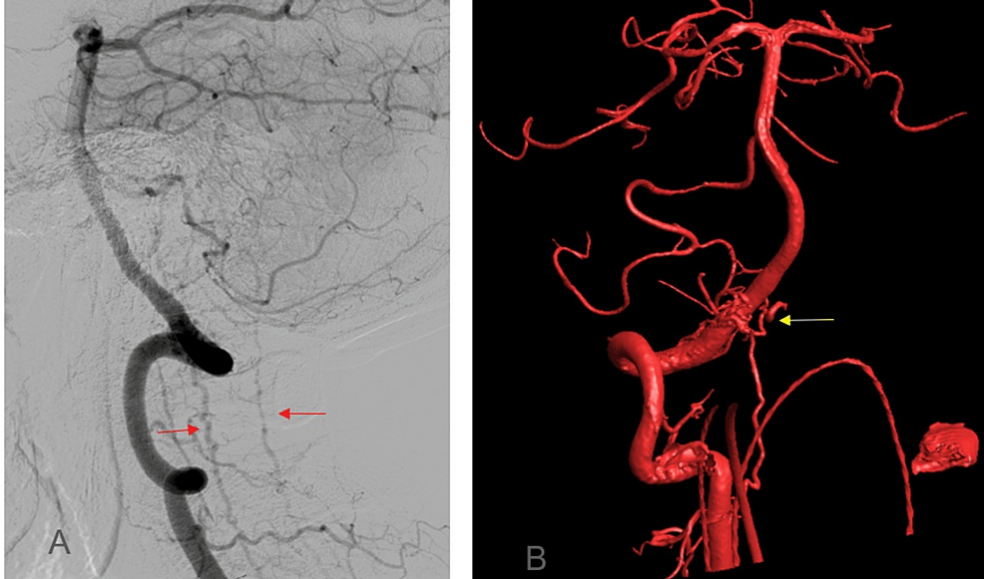
Angiography of the vertebral circulation (A) Right vertebral artery angiogram lateral view, demonstrating shunting into the anterior and posterior spinal veins (red arrows). (B) 3D rotational angiogram in the anteroposterior projection demonstrating small arterial pedicles (yellow arrow) arising directly from the extradural right vertebral artery, providing additional supply to the fistula.

The brain MRI without contrast was done at first to rule out an acute stroke, but it led to a suspicion of an SDAVF. Cervical spine MRI with and without contrast confirmed this diagnosis. Therefore, a neurovascular interventionalist was consulted, who performed an angiogram that is considered the gold standard procedure for diagnosis and treatment. The neurovascular interventionalist performed a cerebral and cervical angiogram. It showed an abnormal arteriovenous shunting seen at the level of the right lateral foramen magnum with arterial supply from the hypoglossal branch of the neuro-meningeal trunk of the right ascending pharyngeal artery, as well as the small unnamed muscular branches of the V3 segment of the right vertebral artery. The venous drainage is exclusively into the anterior and posterior spinal veins (Figures 2, 3).

**Figure 4:**
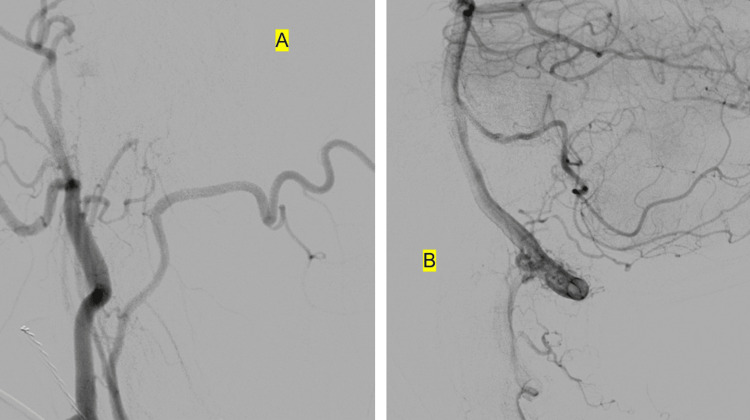
Post-embolization angiography pictures Angiography after embolization of the neuromeningeal trunk of the right external carotid artery using Onyx-18. (A) Lateral view of the right external carotid artery angiogram demonstrating obliteration of the fistula with no supply from the right ascending pharyngeal artery as seen before. (B) Lateral view of the right vertebral artery demonstrating arterial pedicles arising from the extradural right vertebral artery, however, now without shunting into the spinal veins as seen before. These findings confirm the obliteration of the dural AVF.

The provider then performed an angiographic embolization of the dural AVF (Figure 4).

The patient had an incidental COVID-19 infection one day before the embolization happened. She received three days of remdisivir that lasted till one day after the angiogram. She remained on the ventilator after the angiogram for one day and then extubated. Providers placed her on an IV steroid course. She regained neurological function in her extremities and motor power in her left leg. She moved to an acute inpatient rehabilitation facility. Before she left to the rehabilitation facility, her motor exam was as follows: right arm motor power became 5/5, and left arm became 4/5. Motor power was 2/5 for ankle dorsiflexion and plantar flexion in both legs. There was no antigravity movement. There was no evidence of postural, action, or rest tremor. Deep tendon reflexes were 2+ in upper extremities and 1+ in lower extremities symmetrical bilaterally, with the exception of absent ankle reflexes bilaterally. The plantar responses were downward bilaterally.

## Discussion

We present a case with a challenging presentation with an uncommon spinal vascular disease that requires prompt diagnosis and efficient management to prevent progressive spinal function losses. Most SDAVFs are located in the thoracic region, less frequently seen in the lumbar region, and rarely at the cervical level (like our case seen at the cervical level) [3]. It is more male-predominant and usually occurs in middle age. Symptoms could be vague and might progress over months, which would include weakness, motor and sensory loss, gait disturbance, and bowel or bladder dysfunction. Sensory loss could be diffuse or patchy. Provoking factors could be exercise, prolonged standing, change in position, or other routine daily activities like eating [4].

Diagnosis requires a spinal MRI and spinal angiogram. Spinal MRI shows cord edema with central medullary hyperintensity of the conus medullaris and an increase in the volume of the cord, producing the appearance of ascending myelopathy. It also shows a peri-medullary vascular flow with hypointense serpiginous features after contrast agent injection, suggesting venous ectasias. So, diagnosis with MRI relies on high T2 signal or vascular flow voids. Angiography would be diagnostic and therapeutic in this case. It might be challenging given spinal and epidural vascular anatomy variability from one patient to the other. Also, it could carry risks for the patient. For example, the radiculo-meningeal artery could have a segmental medullary branch that would make embolization hazardous because of the significant risk of cord ischemia [5]. 

Treatment could be through endovascular intervention, endovascular embolization, or microsurgery occlusion. The main goal is to block the blood flow through the fistula. Early detection carries a better prognosis for the patient. Late diagnosis is expected in this rare phenomenon, and it could take one to three years before reaching a diagnosis in 10-34% of these cases [6]. Patients who got delayed diagnosis and intervention suffered from complications like spastic or flaccid paralysis or loss of stool and urine continence [6,7]. 

A case series was published of different cases with SDAVF that were misdiagnosed and treated for transverse myelitis or neuromyelitis optica despite the presence of flow voids [8]. MRI could potentially miss the diagnosis. High suspicion should be used when there is unexplained myelopathy, with conus increased signal and vascular flow voids. Spinal Magnetic resonance angiography is more sensitive and less likely to miss the diagnosis [8].

Our case report message points to this rare disease category that could be treated early if well diagnosed, sparing the patient from worsening neurological functional losses that would, unfortunately, happen if the case is not correctly managed.

## Conclusions

Spinal AVF is a rare vascular disease that could affect spinal cord function. Quick diagnosis is essential and requires a high clinical suspicion from different specialties, including physicians from the emergency department, internal medicine department, and neurology department. It also requires prompt referral to a neurovascular interventionalist who would be practicing in tertiary facilities with accessible resources. Treatment could be endovascular embolization or microsurgery occlusion and would require an expert specialist with experience in neuro-vascular procedures to manage the vascular anomaly and achieve accurate results.
